# Within-Host and Between-Host Evolution in SARS-CoV-2—New Variant’s Source

**DOI:** 10.3390/v13050751

**Published:** 2021-04-25

**Authors:** Karin Moelling

**Affiliations:** 1 Institute Medical Microbiology, University Zurich, Gloriastr 30, 8006 Zurich, Switzerland; moelling@molgen.mpg.de; 2 Max Planck Institute for Molecular Genetics, Ihnestr 73, 14195 Berlin, Germany

**Keywords:** corona, pandemic, immunosuppression, emerging variants, risk, origin, prevention

## Abstract

Some of the newly emerging corona viral variants show high numbers of mutations. This is unexpected for a virus with a low mutation rate due to an inherent proof-reading system. Could such a variant arise under very special conditions occurring in a host where the virus replicates and mutates in a rather unlimited fashion, such as in immune compromised patients? The virus was shown to replicate in an immunosuppressed cancer patient for more than 105 days and might be a source of new variants. These patients are asymptomatic and the virus may therefore escape detection and attention and be high-risk. Similarly, HIV-infected individuals may be immunocompromised and support coronavirus replication with increased mutation rates. The patients may promote “within-host evolution”. Some of the viruses present in such a highly mutagenic swarm or quasispecies within one patient may become founders and cause a pandemic by further “between-host evolution”. B.1.1.7 with 23 mutations may be such a case. Immunosuppressed patients can be identified and treated by the synthetic antibody cocktails as passive immunization and kept under control. Immunosuppressed patients can be easily identified and supervised by healthcare workers—once they become aware of the risk—to avoid new variants with pandemic potential.

## 1. Introduction

Viruses mutate. Especially single-stranded RNA viruses have a high mutation rate because their genome is not stabilized by a second matching strand as we know it from the DNA double-helix. It is an inherent viral property and survival strategy to mutate, as it guarantees a large number of progeny viruses even under restrictive conditions. Environmental obstacles can be overcome by escape mutations. Viruses have one major feature: to multiply and generate progeny.

What we know about the single-stranded RNA viruses such as influenza or HIV is not true for coronaviruses—that was the hope of scientists. In contrast to all other known RNA viruses, coronaviruses do not mutate as much. They have a correction system for genome replication, a proofreading enzyme, which removes mismatched nucleotides during replication and transcription [1,2]. The non-structural protein nsp14 encodes an exonuclease which is essential for replication fidelity. If it is deleted, the mutation rates increase 15- to 20-fold, which can become lethal for the virus of this large size [2,3]. This is a consequence of the rather large genome size of the coronaviruses, which is about 30,000 bases long, about 3-fold the size of influenza or HIV RNAs. The size can become dangerous for the survival of the coronavirus, if the replication machinery, mainly the RNA polymerase, which synthesizes the progeny RNA molecules and makes too many mistakes or too many mutations. High error rates could lead to loss of information, the virus could die out. There is a scientific name for it, “error catastrophe”, coined by the Nobel Prize laureate Manfred Eigen [3,4]. He studied evolution using viruses as model. He even suggested to use the principle of increased error frequencies as therapeutic approach against HIV, which did not make it into the clinic. In addition, the proofreading enzyme in coronaviruses hinders the development of antiviral drugs, notably nucleoside analogs which are effective antiviral drugs for disruption of replication of several viruses but failed for coronaviruses.

The error frequency of CoV-2 is about two mutations per month [4]. This is about two to four times lower than for influenza or HIV. Indeed, British scientists from the University College in London published an analysis in *Nature Communications* which found that, among 46,723 isolated and characterized CoV-2 virus genomes from 99 countries, nothing alarming was observed, no mutations associated with increased transmissibility and no indication of viral adaptation to faster spread. Recurrent mutations were evolutionarily neutral and likely induced by the human immune system. There was no matter for concern [5].

## 2. A Previous Mutation

Most people have now forgotten about a mutation which was even briefly described as the “German” mutant, which, although started in China, was then detected in Germany and went around the world almost a year ago [4]. The mutant, D614G, was superior in its replication capacity and displaced the then dominating wild-type within weeks worldwide. It quickly spread from the East to the West Coast of the United States in April 2020 and spread globally [6]. At the position 614 in the spike protein the original D amino acid mutated to G, with the single letter symbols, meaning aspartic acid to glycine, respectively. It replicates faster than the original wild-type and higher titers can be detected in the upper respiratory tract. This manifests itself by the PCR tests, which reach the detection threshold by fewer cycles, indicating higher virus loads. The cycle threshold, Ct, value is lower at higher RNA levels. No increased disease severity was detected and no increase in number of infections could be demonstrated [7,8]. What is most surprising about this mutation is that we still do not exactly know the molecular consequences. One commentary even asked the question: “The coronavirus is mutating—does it matter?” [4]. The mutation occurred early during the pandemic, which might have led to a founder effect, which established a new population. It is located within the surface glycoprotein of CoV-2, the spike protein S, in a region outside of the receptor-binding domain, RBD. The mutation leads to a more “open” conformation of the spike protein S, possibly causing indirectly a higher affinity to the cellular receptor for entry of the virus via the ACE2 receptor in human cells. However, this virus frequently also has three additional mutations, two outside of the S gene and one in the RNA-dependent RNA polymerase (P323L), which may also play a role for the infectivity of this mutant. Some virologists were worried about the appearance of this new mutant such as David Montefiori, a retrovirologist and experienced HIV researcher. This virus is presently still the dominant pandemic strain [7,8].

Most scientists did not expect many further mutations. The virus appeared to be so “fit”, as Charles Darwin defined the success of a species. There did not seem to be a capacity for further increase and selection for higher fitness. No selective pressure or selective advantage seemed to exist to push the virus to change with so many hosts available around the world. However, with so many virus particles spreading around the world, even a slow mutation rate can lead to new viral variants, most of which were characterized by several mutations.

## 3. Newly Emerging Variants

Now, we are witnessing almost a repetition of the previous situation. A new CoV-2 variant was discovered and announced on 4 January 2021, B.1.1.7, which stirred concern and led to shutdowns. This mutant is indeed very highly infectious and may increase the probability of severe disease. It could become a new pandemic.

The B.1.1.7 lineage, also known as N501Y.V1, was first discovered in Kent county in England in December 2020 and within six weeks became the dominant CoV-2 strain in London (Public Health England, 2020). The existence of this variant is very surprising, because, in light of the low mutation rate of coronaviruses, one must find an explanation why it exhibits a total of 17 unique mutations (23 if six synonymous mutations are included that likely have no impact on viral fitness). Six mutations are located in the S protein, which is in total about 1300 amino acids long, with the RBD spanning a region from amino acids 333 to 527 [9]. Not all amino acid changes will be similarly dangerous as the ones in the spike protein. Some of the mutations are even silent, which means they do not lead to any detectable phenotype and we may not notice that easily. Mutations are easier to detect than to understand. The mutant also exhibits deletions, corresponding to omission of regions in the sequence, in total three, including the deletion, H69/V70, which is within the amino terminal domain (NTD) of the S protein distal from the RBD. This could indirectly affect the binding of the virus surface protein to its cellular receptor. The mutation N501Y is located in the RBD of the surface protein leading to a higher affinity to the ACE2 receptor. The increased affinity is thought to cause the higher frequency of infections. This is the most sensitive region of the surface protein and comprises the target of the vaccines against which neutralizing antibodies in vaccines are directed. There is another mutation within the spike protein, P681H, which is immediately adjacent to the furin cleavage site which cleaves the S protein into S1 and S2. Cleavage of the spike protein by the cellular furin protease makes the virus more virulent and increases its ability to enter epithelial cells [10]. This cleavage might be affected by the P681H mutation. Furin has a similar effect on influenza viruses, where the presence of a furin cleavage site in the hemagglutinin surface glycoprotein is associated with increased virulence [11]. 

How can one possibly explain so many mutations and such dramatic changes within one virus isolate?

## 4. Immunosuppression and Within-Host Evolution

Could such a variant with as many as 23 mutations arise under very special conditions occurring in a host where the virus replicates and mutates in a rather unlimited fashion as in the absence of an immune system in an immunocompromised individual? 

A recent paper discusses exactly this, it was published by the American National Institute of Health in the Journal *Cell,* in December of 2020, which describes rather unrestricted virus production in an immunocompromised individual with cancer [12]. Here, a leukemia patient suffering from chronic lymphocytic leukemia (CLL) was described who spent 105 days in a clinical setting with a CoV-2 infection. Yet, he was immunocompromised due to his cancer therapy. The infection did not cause corona-like symptoms, the patient remained asymptotic throughout the course of the infection despite the shedding of infectious CoV-2 for up to at least 70 days. In addition, he did not die of one of the known late stage CoV-2 complications, the cytokine storm, which involves the hyperactivation of the immune system, since he did not have a functioning immune system. The doctors tried to fight against the virus replication with antivirals such as the Ebola drug Remdesivir, which is controversial in its effectiveness and did not have a significant effect. However, one may wonder whether such a drug treatment exerted some selective pressure on the replicating viruses leading to accumulation of mutations. 

The doctors treated the patient with the serum of a patient who had survived the disease and had antibodies in his blood, the convalescent serum. This treatment is an indirect or passive immunization [13,14]. They applied the serum twice and observed slow responses, but finally, succeeded with reduction of the viral load. Perhaps the convalescent serum antibodies were of too low titers or against unmutated virus variants and did not successfully neutralize the type of viruses in the cancer patient. The patient had high virus titers in his upper respiratory tract, his throat. Possibly the antibodies from the serum had little effect there, because the antibodies from the convalescent serum were mainly IgG type and are ineffective within the nasal epithelium and mucosa, where IgA-type immunity is required [13]. The virus isolates were frequently sequenced and genetic variations characterized. The sequences demonstrated a continuous production of viral variants. In immune-competent hosts variations are normally observed at low frequencies. The within-host viral evolution showed among many other mutations some unique variants at the individual timepoints including some deletions [12]. It is unpredictable which of the variants will become dominant and super spreaders. Both spike deletions, observed around day 49 and day 79, around amino acid 140, occurred in the NTD of the S protein, distal from the RBD, a region which is normally not included into modeling of spike structures [15]. Furthermore, the virus did not display altered replication kinetics in vitro. Interestingly, the authors do not alert the readers and do not warn of any danger by the shedding viral variants, but stress that this may occur often unnoticed [12]. The unnoticed production of highly virulent viral variants during a long period of persistent infections could be a real threat. Many variants within one host could also lead to new recombinants, variants with major changes. The authors warn of the asymptomatic appearance of the chronically infected patients, which do not fit to the criteria for frequent PCR testing and may therefore be overlooked. In addition, other rules do not apply, a patient would normally not shed infectious virus for more than two to three weeks. An additional complication with such patients comes from the limit of the PCR tests which recognize viral RNA fragments but do not indicate the presence of replicating infectious viruses [12]. Such spreaders may shed high numbers of virus particles, so there may be the possibility to detect such an excess by a quantitative Real Time (qRT)-PCR, where high viral load allows for low numbers of cycles to reach the cycle-threshold (Ct) and detectable levels. However, this may be difficult to differentiate from acutely infected people with high viral loads and therefore low Ct values. 

Prevention of virus replication could be better achieved by synthetic monoclonal antibody therapy instead of convalescent serum. These are supplied by companies such as Regeneron or Eli Lilly which have received emergency approval for treatment of people early during infection or even as prevention of infection. These synthetic antibody cocktails have proven to reduce the viral load early during infection and for patients undergoing organ transplants or chemotherapy. The reduction of hospitalization and death after early treatment was described as 70% [13,14]. The monoclonal antibodies are similar to the passive immunization sera, now in a modern version as monoclonals. They are effective as long as the virus titer is rather low. At higher titers the dose of synthetic antibodies may not be sufficient. They may be useful to treat long-term coronavirus producers because they may be more potent than convalescent patient sera. Previously, monkey studies with MERS-coronavirus infections showed in an animal model similar results as mentioned here, a similar virus–host dynamic and long-lasting virus shedding after treatment with immune-suppressing drugs such as dexamethasone or cyclophosphamide [12]. 

This patient showed several deletions in the virus isolates. Deletions had also been observed in the pandemic coronavirus from 2003 and correlated with disappearance of the virus and termination of the pandemic [16]. Thus, the loss of genetic information is always a hope for reduced infectivity or disease. However, in this case, the virus did not disappear.

Another report also described an immunocompromised host with persistent viral infection and accelerated viral evolution for 150 days. He was treated with synthetic antibodies leading to three viral reductions and new peaks. He developed 4 new mutations within the spike region and over time 8 additional ones, some disappeared, others were found in structural proteins and the polymerase region [17]. 

Immunocompromised patients may accumulate more mutations than regular patients where the viruses are more constant and may have to be controlled more carefully. High numbers of mutations were also detected in the South African Variant, B.1.351, among them the mutation E484K, which was meanwhile also taken over by the British variant, and there HIV-infected people may also be endangered by some compromised immunity where long-term shedding and altered infection dynamics could result. Recently, an unusual variant with 40 mutations was detected in Angola, where HIV infection rates are high. The South African variants raise the question to what extend HIV/AIDS contribute to their evolution. A potential risk needs to be analyzed. 

**Figure 1:**
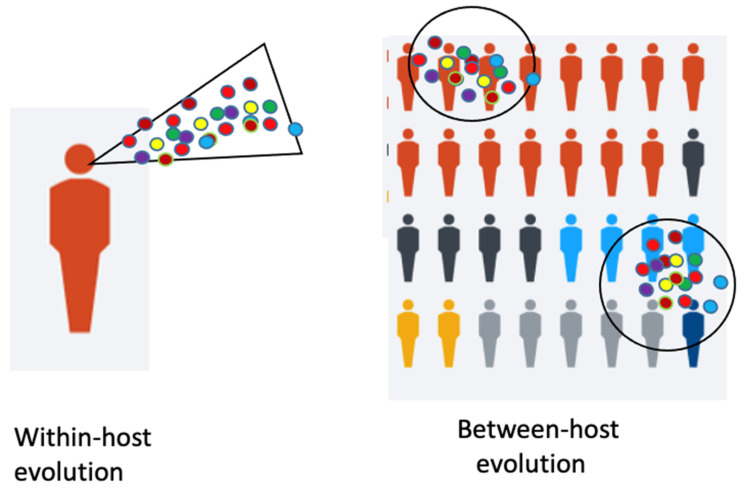
Legend. Within-host and Between-host evolution. De novo mutations of viruses may arise in individual hosts and can become pandemic (modified from [18]).

A model shows the immunocompromised patient with viral “within-host” evolution and many variants (Figure 1).

## 5. Selection of the Fittest

A characteristic property of all viruses is their occurrence as quasispecies, viruses form swarms of closely related members which are, however, distinct. This is due to the above-mentioned error frequency of the polymerase during replication. The high number of mutants may contain members which are more infectious and replication competent than others and will enable one of them to become the winner. In an immune compromised patient, no immune system counteracts the virus, it could freely mutate without immune restrictions. The fastest replicating virus will be the winner. Perhaps B.1.1.7 is such a winner. Furthermore, the selection of the fastest virus is independent of the later disease, which would probably not be of a selective advantage for the virus. 

Immunosuppressed patients are numerous. An estimated 3 million people in the United States have some form of immunocompromising condition, including individuals with HIV infection, solid organ transplant recipients, hematopoietic stem cell transplant recipients and individuals receiving chemotherapy or corticosteroids. The immunocompromised population is furthermore at higher risk of respiratory infections and disease complications [12].

Virologists know from studies in the laboratory that if one grows a virus in cell culture, the viruses will change and mutate and adjust to the experimental or selective conditions. To give an example, in 2015, a coronavirus was engineered in the laboratory with the goal to study viral evolution, determine future emerging viruses and evaluate the disease potential of SARS-like viruses. They adapted the virus to grow efficiently in primary human lung cells with high titers and it gained higher pathogenicity. The research and further studies were restricted by government-mandated pause as too risky. This kind of adaptation is defined as gain-of-function (GoF). It is performed to investigate which mutations could make a virus dangerous and a potential threat. “Further testing in nonhuman primates is required”, but was forbidden and must be outweighed against potential risks [19]. 

The gain of function mutants have previously been generated by two research groups led by Y. Kawaoka and R.A. Fouchier, with influenza virus H5N1 and the goal to define and predict which mutations are potentially dangerous and may give rise to a pandemic. The journals *Nature* and *Science* did not want to publish the details on how to generate such viruses and judged they could be bioweapons. This research was defined as “dual use”, use for learning about dangerous mutants and abuse for bioterrorism as biological weapon. One of the studies was transiently stopped by a moratorium but was ultimately published in reduced form. New regulatory guidelines evaluating risk versus benefits were issued [20,21]. The goal of such studies was to describe what kind of properties would make a virus a candidate for a pandemic virus. 

No more than biosafety level BSL3 laboratories are required for these GoF studies including coronavirus and more potentially pandemic viruses. Only monkey studies require BSL4. These rules should be changed. 

## 6. Antigenic Between-Host Evolution

Meanwhile, as of February 2021, the British variant, characterized by N501Y, has acquired an additional eighths mutation within the spike protein, the same mutation E484K as present in the South African variant B.1.351, V2, which also contains N501Y and others, K417N, N439K-in total nine mutations in the spike region. The mutations E484K and N501Y are important for binding to the ACE2 Receptor. N501Y in the British variant is thought to be responsible for the high infectivity, worldwide fast spread and increased pathogenicity, while the E484K mutation leads to reduction of the effectiveness of some of the vaccines. A third variant in Brazil, P1 or B.1.1.248, also contains these two mutations and K417T in the spike region and in total 17 mutations [22,23].

In the city of Manaus, Brazil, achievement of herd immunity had been assumed with about three-quarters of the population seroconverted. This was estimated as above the theoretical threshold for herd immunity (60–67%) [23,24]. For several months the infection rate was low but then exploded. Was it caused by escape mutations or incorrectly calculated herd immunity and much lower infection rates? The interpretations are controversial. Explanations for the extensive second wave of transmission also comprise possible waning of the antibody titers, socioeconomic conditions, crowded households, poor hygiene, multiple imported infections and mobility including river boat transportations [23]. The situation in Manaus does not allow a general conclusion about a successful herd immunity. P1 accumulated ten mutations in the spike region, including N501Y, known from the British variant B.1.1.7 and, again N501Y, E484K, K417N/T known from the South African variant B.1.325, whereby the E484K mutation most strongly reduces neutralization by antibodies, which would support escape mutations.

In addition, in southern California a new mutant showed up, CAL.20C, which is spreading faster and may cause more severe disease that the wild-type virus. It was first detected in July 2020 in Los Angeles and from September to January increased to a prevalence of above 50% in California. It also occurs in other States of the US. It is more infectious, associated with more severe illness and partially resistant against neutralizing antibodies. It is a variant “of concern”. There are two forms, B.1.427 and B.1.429 with slightly different mutations, both are also referred to as 20C/L452R. It has three mutations, one of them being L452R in the RBD. None of these three spike mutations is found in the three other main variants, from the UK, South Africa and Brazil [24,25]. 

Furthermore, a new variant appeared in New York recently described on Feb. 23rd, 2021, B.1.526 with seven spike mutations (L5F, T95I, D253G, E484K or S477N, D614G and A701V) [26].

The new Indian variant B.1.617 first detected in Oct 2020 and in Feb. 2021 in England, has 13 mutations, two of them in the RBD such as E484Q and L452R.

Some of the mentioned variants exhibit some identical mutations. It is assumed that they arose independently. These mutations in the RBD and other regions must have been independently selected for by their higher fitness, increased virus reproduction rates and provided a selective advantage by escape from the immunity of a host against a previous variant. This raises some hope, that the various escape mutations are identical. These variants are either spontaneous mutants, due to “between-host evolution”, or they are escape mutations (Figure 1). In contrast to the immunosuppressed patients, who can become super-spreaders of different variants during their long period of chronic infections by “within-host evolution”, the escape mutations arise under selective pressure from the environment, the host immunity. As detected in Manaus, the herd immunity may have been overestimated [24]. A few people were infected a second time; thus, superinfection could occur and overcome immunity but perhaps prevent severe disease. The high virus titers may have developed by virus evolution with new variants, some of which escaped from the hosts’ immunity. What we witness is antigenic evolution of high-titer virus which may occur independently of the mutation rate of the virus. Furthermore, the immune system still works against its initiator identical strain, but less so against a mutant strain, depending on the mutations. Thus, a waning immunity is not the main or only cause of the new virus emergence. 

There is some immunity even against the common seasonal coronaviruses in the population, against the four coronaviruses we encounter every winter. Immunological memory after infection with seasonal human coronaviruses was shown to contribute to cross-protection against CoV-2 [18]. This is also the case with influenza viruses, new vaccines are necessary every winter season because of antigenic drift of the virus. The existing immunity can last for much longer. The yearly new vaccines are necessary because the virus escapes the immunity against the previous strains of there are too many mutations [18].

## 7. Influenza as Model?

Even though influenza and coronaviruses differ in many respects, they share some features. They differ in duration of the disease and the onset of the disease after infection. However, similarly, the influenza viruses evolve rapidly at a global scale. Interestingly, Xue et al. [27] from Seattle, describe that this evolution begins with mutations within an infected host, which is described as within-host evolution. These newly emerging variants are the beginning of a global evolution. One must distinguish between two kinds of viral evolution: one within a host and the other one between hosts (Figure 1). Each influenza infection generates a cloud or swarm (quasispecies) of diverse viral variants by mutations. Factors such as antigenic selection, antiviral treatment, drugs, tissue specificity, spatial structure and multiplicity of infection influence how a virus evolves within a host. 

Influenza virus infections typically last for 5–7 days. Acutely infecting viruses peak with viral production after 2–3 days and this is a short period to develop many mutations. Higher diversity can be caused by infection with multiple related viral strains. Chronic infections, in contrast, can favor extensive evolution and antigenic variants can reach high within-host frequencies. In addition, drug-resistant variants may arise. An important parameter for intra-host variations may also be attributed to the initial viral dose, the multiplicity of infection (MOI). The new variants may become founders of one or several global variants. The within-host arising variations may parallel global evolutionary trends.

Beginning in December 2020, the van Dorp group at the University College, London, mentioned above, found 12,706 mutations in CoV-2, 398 of these occurring frequently and repeatedly and 185 have occurring at least three times. The authors ask whether they happened to occur and what the chance may be for higher transmission by a so-called founder effect, leading to more efficient transmission and possibly the start of a pandemic. To them, this did not appear to be the case [5]. The information on influenza can be applied, to some extent, to coronavirus infections, the within-host evolution and the between-host evolution. Even the long period of asymptomatic infection in coronavirus hosts without immunosuppression could allow a within-host virus evolution. This type of evolution is stronger in immunocompromised patients. These patients could initiate spreading of variants in dense populations, as suggested by the high number of mutations, which would not be expected by between-host viral evolution. Between-host evolution will favor escape mutants, evading the immunity of the host as in Manaus or in South Africa and possibly also with the new Indian variant. 

We may have to become more aware of newly emerging variants and we may have to watch out specifically for immunosuppressed people and try to reduce the risk of evolution of new virus variants in such specific cohorts. A selected number of people hot-spots, rather than random screening, would be sufficient watch out for these; they need a focused observation, testing and follow-up. 

The newly emerging viruses can be detected by new software tools, e.g., the Variant Data Base (VDB) founded at the California Institute of Technology, with a focus on the spike sequence [18]. Perhaps, then, we may be faster than the virus, ahead of newly arising variants, not behind, as we currently are.

## Data Availability

Not applicable.
